# From everywhere all at once: Several colonization routes available to Svalbard in the early Holocene

**DOI:** 10.1002/ece3.9892

**Published:** 2023-03-19

**Authors:** Viktorie Brožová, Johannes S. Bolstad, Alexey P. Seregin, Pernille B. Eidesen

**Affiliations:** ^1^ Department of Botany, Faculty of Science University of South Bohemia in České Budějovice České Budějovice Czech Republic; ^2^ Department of Arctic Biology The University Centre in Svalbard Longyearbyen Norway; ^3^ Herbarium (MW), Faculty of Biology M. V. Lomonosov Moscow State University Moscow Russia; ^4^ Department of Biosciences University of Oslo Oslo Norway

**Keywords:** Arctic, diversity hotspot, haplotype, Holocene, phylogeography, Svalbard

## Abstract

For many arctic species, the spatial (re‐)colonization patterns after the last Pleistocene glaciation have been described. However, the temporal aspects of their colonization are largely missing. Did one route prevail early, while another was more important later? The high Arctic archipelago Svalbard represents a good model system to address timeframe of postglacial plant colonization. Svalbard was almost fully glaciated during last glacial maximum and (re‐)colonization of vascular plants began in early Holocene. Early Holocene climatic optimum (HCO) supported an expanded establishment of a partly thermophilic vegetation. Today, we find remnants of this vegetation in sheltered regions referred to as “Arctic biodiversity hotspots”. The oldest record of postglacial plant colonization to Svalbard is found in Ringhorndalen‐Flatøyrdalen. Even though thermophilic species could establish also later in Holocene, only HCO was favorable for vast colonization, and only hotspots offered stable conditions for thermophilic populations throughout Holocene. Thus, these relic populations may reflect colonization patterns of HCO. We investigate whether the colonization direction of thermophilic plants (*Arnica angustifolia*, *Campanula uniflora*, *Pinguicula alpina*, *Tofieldia pusilla*, and *Vaccinium uliginosum* ssp. *microphyllum*) in Ringhorndalen‐Flatøyrdalen was uniform and different from later colonization events in other localities and non‐thermophilic plants (*Arenaria humifusa*, *Bistorta vivipara*, *Juncus biglumis*, *Oxyria digyna*, and *Silene acaulis*). We analyzed plastid haplotypes of the 10 taxa from Ringhorndalen‐Flatøyrdalen, from later‐colonized localities in Svalbard, and from putative source regions outside Svalbard. Only rare and thermophilic taxa *Campanula uniflora* and *Vaccinium uliginosum* ssp. *microphyllum* provided results suggesting at least two colonization events from different source regions. *Tofieldia pusilla* and all the non‐thermophilic plants showed no clear phylogeographically differentiation within Svalbard. Two of the thermophilic species showed no sequence variation. Based on the results, a uniform colonization direction to Svalbard in early Holocene is not probable; several source areas and dispersal directions were contemporarily involved.

## Graphical abstract

The spatial patterns of Holocene recolonization of Svalbard have been thoroughly described; the temporal aspect is, however, largely missing. Did one direction prevail at the beginning of Holocene while another was more important later? Based on the plastid haplotype data of thermophilic and non‐thermophilic taxa, a uniform colonization direction to Svalbard in early Holocene is not probable; several source areas and dispersal directions were contemporarily involved.

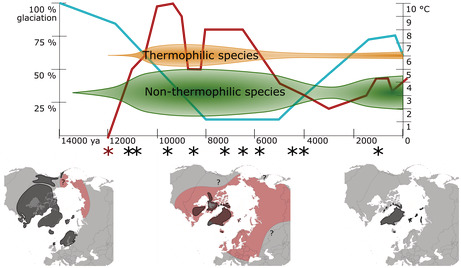

## 1 INTRODUCTION

**FIGURE 1:**
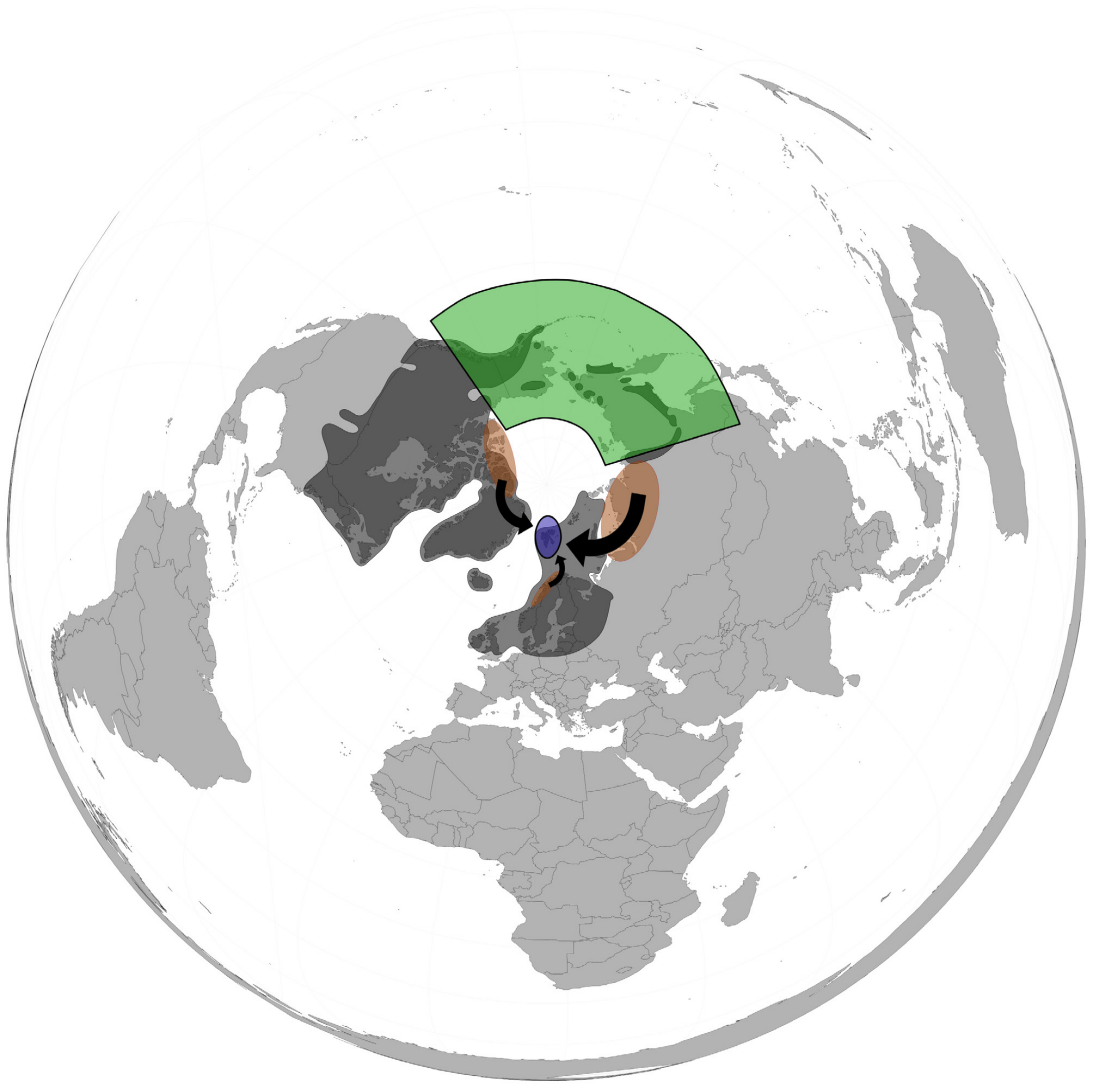
Last glaciation maximum (LGM) and dispersal pathways in Svalbard. Dark gray – LGM glacier extent; green – area of Beringia (Hultén, 1937); blue – position of Svalbard; orange – dispersal source areas for Svalbard; arrows thickness corresponds with importance of the source area (Alsos et al., 2007).

The distribution of current Arctic plant diversity is a result of climatic changes during Pleistocene (2.5–0.01 Mya) when vast areas of the Arctic were recurrently fully glaciated (Ehlers & Gibbard, 2007). Due to glaciations, distribution ranges were shifted and fragmented. During interglacials, ranges were (re‐)established by colonization from various refugia along the ice margins. The last wave of (re‐)colonization started after the last glacial maximum (LGM; 26.5 to 19 kya depending on site; Dyke, 2004; Hughes et al., 2016; Young & Briner, 2015), and in some regions, deglaciation and colonization are still ongoing (Figure 1). The spatial patterns of the post‐Pleistocene recolonization have been thoroughly described; the temporal aspect of Holocene colonization is, however, largely missing (but see Ikeda et al., 2017). Did colonization routes change through time? Did one direction prevail at the beginning of the colonization, while another was more important later? Connecting available knowledge about Pleistocene glacial history, Holocene colonization patterns, and ancient and recent vegetation composition may shed light on temporal variation during (re‐)colonization.

In general, the current plant diversity in a given Arctic region is strongly related to the level of climatic stability in the region throughout the Pleistocene. Glacial refugia served as the most stable areas; the majority of them were however only temporary or only of local importance (summarized in e.g., Abbott & Brochmann, 2003 or Hewitt, 2004). One of the most stable Arctic regions is the area around the Bering Strait, called Beringia (Hultén, 1937). During glaciations, the Bering Strait was dry land connecting continents through a land bridge, whereas the surrounding areas remained unglaciated. Thus, Beringia served as an important Arctic refugium and biodiversity source throughout the Pleistocene (Hultén, 1937). Today, Beringia is characterized by high species richness, high levels of endemism, high levels of genetic diversity and ancestral genotypes (Abbott & Brochmann, 2003; DeChaine, 2008; Yurtsev, 1982).

**FIGURE 2:**
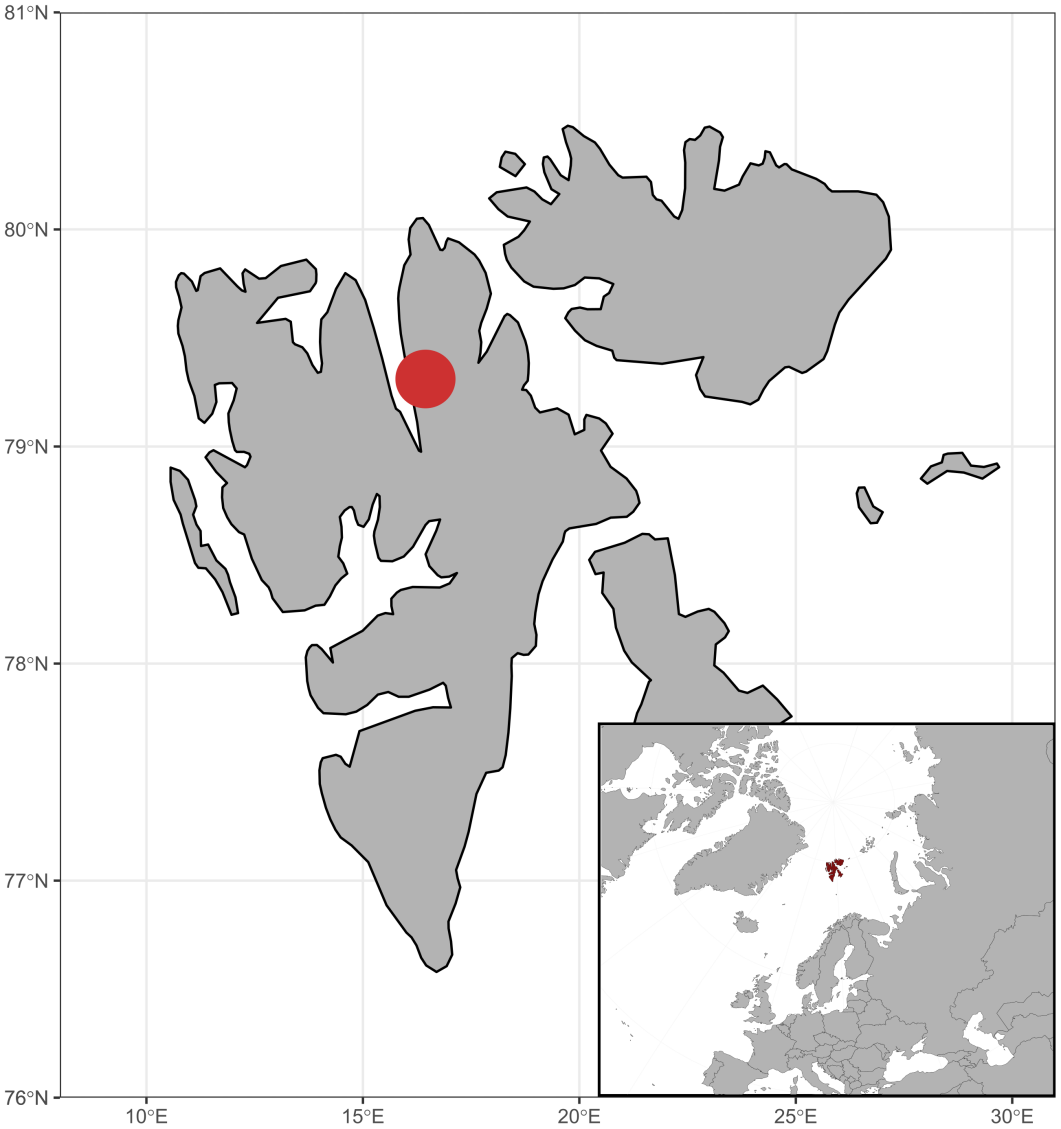
Map of Svalbard and its position in the Arctic. Ringhorndalen‐Flatøyrdalen hotspot is highlighted by the red point.

**FIGURE 3:**
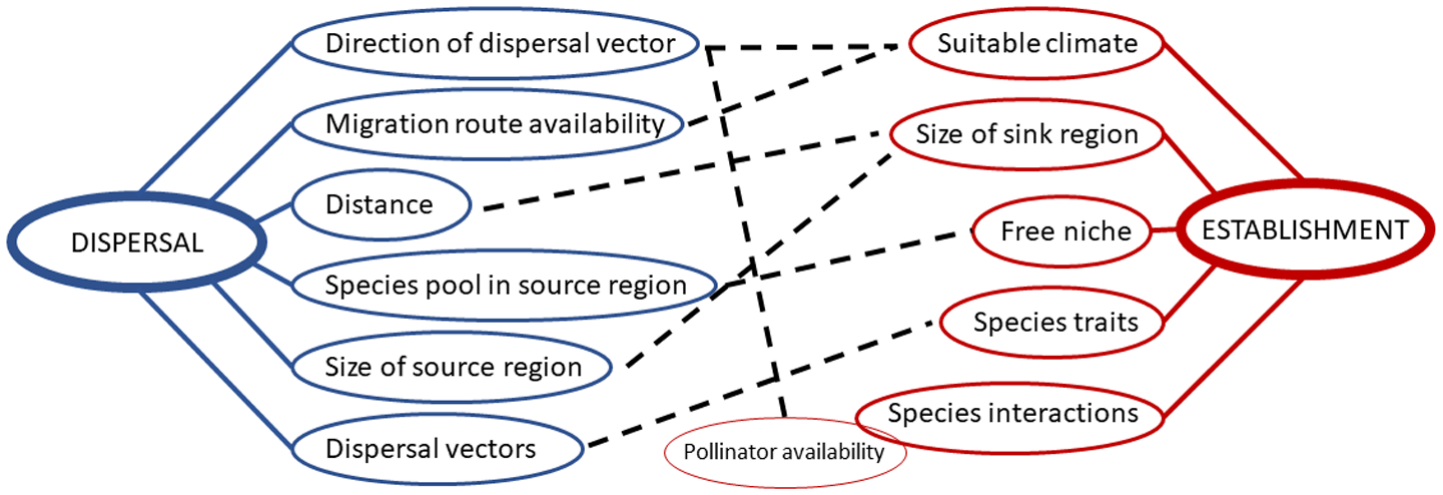
Dispersal and establishment network. Factors associated with dispersal and establishment are connected to each of the aspects by full line. Dashed lines show dependence of individual factors on each other.

In contrast to Beringia, the high Arctic archipelago Svalbard (74°20′–80°50′ N, 10°30′–33°30′ E, Figure 2), situated at the northernmost fringe of the Atlantic Ocean, was heavily glaciated (e.g., Hughes et al., 2016; Landvik et al., 1998), and the current flora largely colonized the archipelago during Holocene (ca. 10 kya – present). Thus, the current biodiversity of Svalbard, from gene to ecosystem level, is comparatively low (Eidesen, Ehrich, et al., 2013). This pattern is further reinforced by its remote location, surrounded by vast oceans which represent major dispersal barriers (Eidesen, Ehrich, et al., 2013). In Svalbard, natural dispersal and establishment of new plant species is likely an ongoing process (Alsos et al., 2007; Coulson, 2015; Ware et al., 2012) which depends on many biotic and abiotic factors (Figure 3). The fact that Svalbard has a set timeframe for colonization, and any colonization event must happen from a distant source area leading to some level of funder effect, has made Svalbard a perfect area to study postglacial colonization by long‐distance dispersal (LDD; Alsos et al., 2007).

Several phylogeographical studies concerning plant colonization of Svalbard during Holocene have been published in the last decades (e.g., Abbott et al., 1995; Alsos, Ehrich, et al., 2015; Alsos, Ware, et al., 2015; Alsos et al., 2007; Birkeland et al., 2017; Gabrielsen et al., 1997; Westergaard et al., 2010, 2011). The current flora clearly originated from several source regions (i.e., not in the meaning of a refugium, but in the meaning of a land deglaciated earlier than Svalbard serving as the last steppingstone before colonizing Svalbard). Siberia has been the most important source region despite being the most distant one (Alsos et al., 2007). This is probably a consequence of Taimyr and Yamalo‐Nenets regions remaining largely unglaciated during LGM and at the same time keeping connection with Svalbard by sea ice for a long period (CLIMAP Project, 1981). A western pathway of colonization is also shown to be an important source of propagules, while colonization from southern source regions such as Scandinavia appears less common (Alsos et al., 2007). We assume that all these source localities for the Svalbard flora were situated along the northern coasts of Siberia, Scandinavia, Greenland, and Canada which are suggested to be partly unglaciated at the end of the last glaciation (Briner et al., 2016; Hughes et al., 2016; Young & Briner, 2015). This northern route along the coast represents the shortest and probably easiest way where plants could spread directly over sea ice (Figure 1).

Additional to the size and position of available source regions, understanding characteristics of putative dispersal vectors may inform about both temporal and spatial aspects of long‐distance plant migration routes (e.g., Muñoz et al., 2004; Nathan, 2006; Robledo‐Arnuncio et al., 2014; Sauer, 1988). Plant colonization of Svalbard is dependent on LDD by chance (Carlquist, 1981) which may be aided by various dispersal vectors, such as wind, sea ice (Alsos, Ehrich, et al., 2015), and driftwood (e.g., Dyke et al., 1997; Eggertsson, 1994). These vectors are influenced by oceanographic currents and wind patterns which may change through time (Dyke et al., 1997; Moros et al., 2004; Tremblay et al., 1997). Even at present, west and north coasts of Svalbard are influenced by different sea currents (Loeng & Drinkwater, 2007). Thus, the importance of different dispersal vectors has likely shifted during Holocene which again may have influenced the level of dispersal from different source areas over time.

**FIGURE 4:**
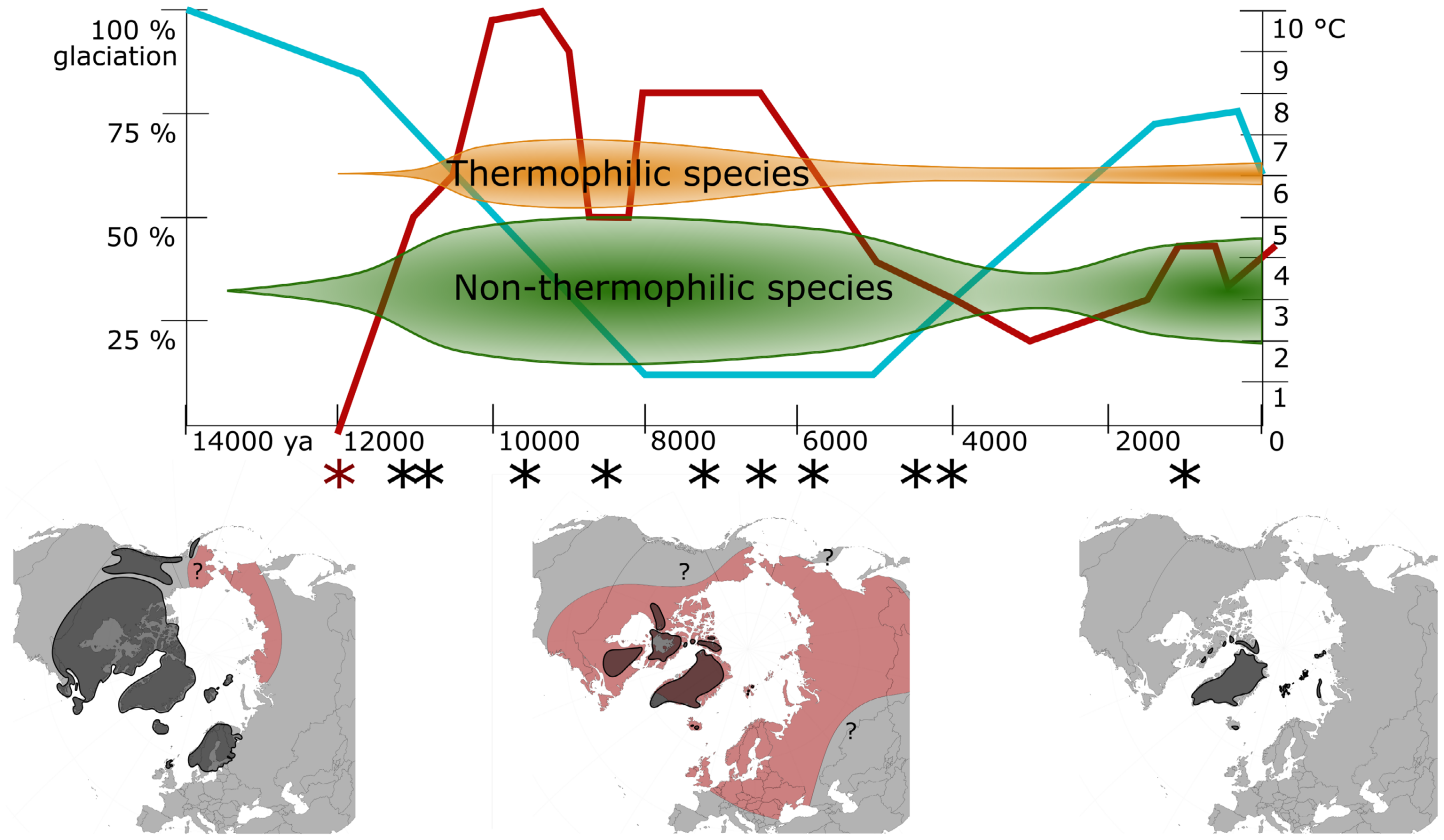
Environmental conditions in Svalbard and the Arctic during Holocene. The time scale spans from 14 kya until recent. Blue line – extent of glaciation in Svalbard (Farnsworth et al., 2020); red line – average summer temperature in Svalbard (Mangerud & Svendsen, 2018); orange – timeframe of potential thermophilic species establishment; green – timeframe of potential non‐thermophilic species establishment (the sizes of the establishment timeframes illustrate our hypothesis); asterisk – vegetation age datings (summarized by Bernardová & Košnar, 2012; Hyvärinen, 1970; Alsos et al., 2016); red asterisk – vegetation age in Ringhorndalen (Voldstad et al., 2020); in maps: gray – glacier extent (Dyke, 2004; Farnsworth et al., 2020; Hughes et al., 2016), red – temperature higher than today (Davis et al., 2003; Edwards et al., 2001; Velichko et al., 2002; Zhang et al., 2010), question marks – uncertainty about temperature.

Colonization is not only dependent on dispersal from source regions; suitable establishment conditions in sink regions are of equal or even higher importance (Alsos et al., 2007; Figure 3). During Holocene, the climatic conditions in Svalbard changed drastically (Farnsworth et al., 2020; Mangerud & Svendsen, 2018; Figure 4). The warmest and most favorable climatic conditions occurred just after deglaciation. This period is called the Holocene climatic optimum (HCO). The peak of HCO in Svalbard, with the average July temperature 6°C higher than today, has been dated to 10.2–9.2 cal. kya (Alsos et al., 2016; Birks, 1991; Birks et al., 1994; Hald et al., 2004; Hyvärinen, 1970; Mangerud & Svendsen, 2018; Miller et al., 2010; Salvigsen et al., 1992; Salvigsen & Høgvard, 2006; Svendsen & Mangerud, 1997). Other regions of the Arctic experienced HCO later (Bezrukova et al., 2011; Blyakharchuk & Sulerzhitsky, 1999; de Vernal et al., 2005; Gajewski, 2015). During HCO, the glacial extent was at its minimum, and large areas were available for colonization. After HCO, there was a cooler period followed by a slight warming, less intense than the first one (Mangerud & Svendsen, 2018). After 6 cal. kya, the climate gradually cooled toward the present temperatures. This climatic progression was the main factor controlling population establishment possibilities in Svalbard; that is, thermophilic species had optimal conditions for vast establishment only during the most favorable periods (Alsos et al., 2016; Voldstad et al., 2020). A recent study, which mapped time of plant colonization in Fennoscandia based on *seda*DNA, showed that adaptation to temperature had the highest relative predictive power to estimate time of colonization (Alsos et al., 2022). Thus, we assume that even though populations of thermophilic species could have established occasionally in certain locations during the whole Holocene, most of them have established when climatic conditions in the locality allowed it. We further assume that populations in climatically stable hotspots could have persisted to present day while populations in less stable localities most likely disappeared during colder periods. On the contrary, more cold‐tolerant species could manage to establish and survive anytime during Holocene.

In our study, we focus on Ringhorndalen‐Flatøyrdalen area (Figure 2), the most diverse and isolated biodiversity hotspot in Svalbard (Birkeland et al., 2017; Eidesen, Strømmen, et al., 2013; Elvebakk & Nilsen, 2002, 2016). It hosts 124 vascular plants species out of 204 taxa recorded from Svalbard with some of them having the only known location in Svalbard here (*Pinguicula alpina*, *Luzula spicata*, *Erigeron uniflorus*, *Draba* aff. *oblongata*, *Festuca ovina*, and *Calamagrostis purpurascens*; Eidesen, Strømmen, et al., 2013; Eidesen et al., 2018; Elvebakk & Nilsen, 2002, 2016). This area is one of four Svalbard hotspots where conditions for plant growth become more favorable than average resulting in higher productivity and/or species diversity (Elvebakk, 2005; Walker, 1995). Ringhorndalen‐Flatøyrdalen shows evidence of early postglacial colonization with stable and favorable environmental conditions throughout Holocene offering suitable conditions for relic thermophilic vegetation from the HCO (Voldstad et al., 2020). Recent evidence suggests that this area was opened for colonization as early as 12.0 kya (Voldstad et al., 2020), which is earlier than other investigated localities (Figure 4; summarized by Alsos et al., 2016; Bernardová & Košnar, 2012; Hyvärinen, 1970). We aim to utilize the specific features of this locality and compare the genetic composition of populations in Ringhorndalen‐Flatøyrdalen with other populations in Svalbard, and putative source populations outside Svalbard.

As this area was opened early for colonization and remained stable, it has kept the biodiversity of early established thermophilic populations throughout HCO and the cooler periods too. Thus, current genetic signatures of hotspot thermophilic species most likely represent descendants from lineages colonizing Svalbard before or during the HCO. Their current fragmented distribution ranges restrict gene flow among populations within Svalbard. Thermophilic species in hotspots in general, and in the early deglaciated area Ringhorndalen‐Flatøyrdalen in particular, might therefore reveal information about source area(s) or dispersal vectors important in early Holocene.

To validate the expected pattern of rare, thermophilic species in the Ringhorndalen‐Flatøyrdalen area, we built a comparative dataset of common, non‐thermophilic species in Svalbard. We predict that non‐thermophilic species show little temporal correlation, higher levels of genetic diversity, and genetic mixture, with lineages originating from several source areas compared to rare thermophilic species. Moreover, since non‐thermophilic species are widely distributed and less limited by temperature for successful establishment, we expect higher gene flow within Svalbard. Thus, we do not expect to find clear genetic differences between populations of common, non‐thermophilic species from the Ringhorndalen‐Flatøyrdalen area and other localities.

We predict that if populations of thermophilic species in Ringhorndalen‐Flatøyrdalen show a coherent direction of dispersal, whereas other localities colonized later and less thermophilic species show a larger mixture, main direction of dispersal have likely changed through time. More specifically, we predict that (1) if source regions and/or direction of dispersal are coherent among thermophilic species in Ringhorndalen‐Flatøyrdalen, but not coherent among other hotspot areas in Svalbard, there was one dominating source/direction of dispersal in early Holocene, but a shift in source/direction occurred before the end of the HCO; (2) if source regions and/or direction of dispersal are coherent among thermophilic species in Ringhorndalen‐Flatøyrdalen and the other hotspot localities, there was one dominating source/direction until the end of the HCO; (3) if there is no coherent pattern among thermophilic species, several sources/direction of dispersal were contributing already at an early stage of colonization.

To evaluate these predictions, we used a comparative phylogeographic approach including 10 species: five thermophilic (*Arnica angustifolia*, *Campanula uniflora*, *Pinguicula alpina*, *Tofieldia pusilla*, *Vaccinium uliginosum* ssp. *microphyllum*) and five non‐thermophilic species (*Arenaria humifusa*, *Bistorta vivipara*, *Juncus biglumis*, *Oxyria digyna*, and *Silene acaulis*) with wide distributions outside Svalbard. For six species, we compiled previously published large‐scale phylogeographies based on haplotype diversity (sequences of non‐coding plastid DNA regions; Allen et al., 2012; Alsos et al., 2005; Eidesen, Alsos, et al., 2007; Gussarova et al., 2015; Marr et al., 2013; Schönswetter et al., 2007; Wang et al., 2016; Westergaard et al., 2011) with additional samples from Svalbard and Ringhorndalen‐Flatøyrdalen. For four species, we built large‐scale phylogeographies from scratch.

## 2 MATERIALS AND METHODS

### 2.1 Selection of species

We selected 10 taxa, five thermophilic and five non‐thermophilic, based on the following criteria: wide Arctic distribution range (several possible source areas for colonization of Svalbard), availability of phylogeographic data (only phylogeographies based on plastid DNA haplotype data were chosen to enable extension of dataset), and presence of species in Ringhorndalen‐Flatøyrdalen area. Taxa with available phylogeographies meeting these criteria included five non‐thermophilic (*Arenaria humifusa* Wahlenb. in Westergaard et al., 2011; *Bistorta vivipara* (L.) Delarbre in Marr et al., 2013; *Juncus biglumis* L. in Schönswetter et al., 2007; *Oxyria digyna* (L.) Hill. in Allen et al., 2012; Wang et al., 2016; and *Silene acaulis* (L.) Jacq. in Gussarova et al., 2015) and one thermophilic subspecies (*Vaccinium uliginosum* L. ssp. *microphyllum* (Lange) Tolm., Alsos et al., 2005; Eidesen, Alsos, et al., 2007). Data for these six taxa were downloaded from GenBank (https://www.ncbi.nlm.nih.gov/). For *Arenaria humifusa*, sequences with accession numbers HM772095, HM772096, HM772106 – HM772108 were used, for *Bistarta vivipara* JX853580.1‐JX853609.1 were used, for *Juncus biglumis* AM085712.1‐AM085736.1 were used, for *Oxyria digyna* JQ080918.1‐JQ080962.1 and KR003612.1‐KR003628.1 were used, and for *Vaccinium uliginosum* DQ073105‐DQ073326 and EF502102‐EF502167.

Selection of the other four thermophilic species without available phylogeographies based on plastid DNA haplotypes (*Arnica angustifolia* Vahl, *Campanula uniflora* L., *Pinguicula alpina* L., *Tofieldia pusilla* (Michx.) Pers.) was based on the same distribution criteria and the availability of silica samples (from Ringhorndalen‐Flatøyrdalen area and from Svalbard in general).

#### 2.1.1 Thermophilic species

The five thermophilic species (*Arnica angustifolia*, *Campanula uniflora*, *Pinguicula alpina*, *Tofieldia pusilla*, *Vaccinium uliginosum* ssp. *microphyllum*) are rather rare with scattered occurrences in Svalbard. They typically are found in favorable, stable, and warmer sites, but have different habitat preferences (Table 1). *Campanula uniflora*, *Pinguicula alpina*, and *Tofieldia pusilla* are red‐listed as Near Threatened, and *Vaccinium uliginosum* ssp. *microphyllum* as Critically Endangered (https://svalbardflora.no).

**TABLE 1:** Ecological, reproductive, and conservation characteristics of species used in the study.

Taxon	Family	Thermophilic/non‐thermophilic	Habitat	Dispersal adaptation	Pollination	Red‐list	Existing large‐scale phylogeography
*Arenaria humifusa* Wahlenb.	Caryophyllaceae	Non‐thermophilic	Less vegetated spots, gravel, frost patterned ground, or disturbed moist heath	Seeds – along the ground	Self‐pollination	VU	Westergaard et al. (2011)
*Bistorta vivipara* (L.) Delarbre	Polygonaceae	Non‐thermophilic	Common in a broad range of habitats except for tall‐grown vegetation	Bulbils – spread by water and by herbivores	–	–	Marr et al. (2013)
*Juncus biglumis* L.	Juncaceae	Non‐thermophilic	In moist and wet habitats with little competition	Seeds – spread by wind, water, or by animals	Wind, self‐pollination	–	Schönswetter et al. (2007)
*Oxyria digyna* (L.) Hill.	Polygonaceae	Non‐thermophilic	Moist or wet habitats, moderately vegetated sites, common under bird cliffs	Winged seeds adapted to wind‐dispersal	Wind	–	Allen et al. (2012), Wang et al. (2016)
*Silene acaulis* (L.) Jacq.	Caryophyllaceae	Non‐thermophilic	Moderately to densely vegetated heaths, and snow beds	Seeds – spread by wind, water, or by animals	Insect	–	Gussarova et al. (2015)
*Arnica angustifolia* Vahl	Asteraceae	Thermophilic	Dry grassy heaths and meadows, stabile vegetation cover, on slopes and on cliff ledges	Light seeds with hairs adopted for wind distance	Asexual	LC	–
*Campanula uniflora* L.	Campanulaceae	Thermophilic	Dry grassy heaths and meadows, stabile vegetation cover, on slopes and on cliff ledges	Seeds – ballistic dispersal by wind and by animals	Insect, self‐pollination	NT	–
*Pinguicula alpina* L.	Lentibulariaceae	Thermophilic	Shallow mires with sedges and rushes, or in dense tussock vegetation of *dryas octopetala*, *cassiope tetragona*, and mosses	Seeds – ballistic dispersal by wind and by animals	Insect	NT	–
*Tofieldia pusilla* (Michx.) Pers.	Tofieldiaceae	Thermophilic	Shallow mires with sedges and rushes, or in dense tussock vegetation of *dryas octopetala*, *cassiope tetragona*, and mosses	Seeds – ballistic dispersal by wind and by animals	Self‐pollination	NT	–
*Vaccinium uliginosum* L. ssp. *microphyllum* (Lange) Tolm.	Ericaceae	Thermophilic	Slopes in well‐developed dense heath vegetation	Barriers adapted to birds and mammals zoochory	Insect, self‐pollination	CR	Alsos et al. (2005), Eidesen, Alsos, et al. (2007)

#### 2.1.2 Non‐thermophilic species

Four out of five non‐thermophilic species are widely distributed in Svalbard (*Bistorta vivipara*, *Juncus biglumis*, *Oxyria digyna*, and *Silene acaulis*), but have different dispersal adaptations (Table 1). *Arenaria humifusa* is not regarded as thermophilic but is rare in Svalbard and red‐listed as Vulnerable (https://svalbardflora.no). Ringhorndalen‐Flatøyrdalen is one of the few locations it is found in Svalbard.

### 2.2 Sampling of material

Samples for DNA sequencing of all species were either collected in the field by us, extracted from herbarium vouchers, or obtained from private collections (Table S1). Specifically, samples from Ringhorndalen were collected in years 2015 and 2019 during field camps. Samples from Kongsfjorden and Krossfjorden were collected in summer 2020 during a field camp. In the field, we collected green leaf material into silica‐gel for fast drying of the tissue. We collected 1–5 individuals per taxon, always at least 5 m apart.

Samples from the other Svalbard localities and from the rest of the Arctic were obtained from herbarium collections in Natural History Museum in Oslo (O), Arctic University Museum of Norway (TROM), Canadian Museum of Nature (CAN), and Natural History Museum of Denmark (C). Samples of *Tofieldia pusilla* from Europe were obtained from private collection of Siri Birkeland.

### 2.3 DNA extraction and sequencing

DNA was extracted from dry leaf material using DNeasy Plant Mini Kit (Qiagen) following the standard protocol. For each species, one to three individuals from Ringhorndalen‐Flatøyrdalen population were sequenced; one individual per population was sequenced for the other localities. For species with existing phylogeographical dataset, regions used in the original study were sequenced (Allen et al., 2012; Alsos et al., 2005; Eidesen, Alsos, et al., 2007; Gussarova et al., 2015; Marr et al., 2013; Schönswetter et al., 2007; Wang et al., 2016; Westergaard et al., 2011). For the other species, eight different plastid non‐coding regions were tested to search for variable regions: *rps*16‐*trn*Q, *rpl*32‐*trn*L (Shaw et al., 2007), *psb*A‐*trn*H (Sang et al., 1997), *pet*B‐*pet*D (Löhne & Borsch, 2005), *trn*L‐*trn*F (Taberlet et al., 1991), *trn*S‐*trn*G (Shaw et al., 2005), *trn*T‐*trn*L (Cronn et al., 2002), and *ycf*4‐*cem*A (Ekenäs et al., 2007).

For 25 μL PCR reaction, we used 0.2 μL DreamTaq polymerase (5 U/μL; Thermo Scientific), 0.5 μL dNTPs (10 mM), 0.5 μL of each primer (10 μL), 2.0 μL MgCl_2_ (25 mM), 2.5 μL 10× PCR buffer, and 3.0 μL template DNA. PCR cycling was performed with a Mastercycler X50 (Eppendorf) with specific parameters for each primer pair (Table 2). Sequencing was performed by Eurofins genomics (Germany) and stored in GenBank (Table S1).

**TABLE 2:** PCR programs used for selected pDNA regions.

	*trn*S‐*trn*G, *rpl*32‐*trn*L, *rps*16‐*trn*Q		*trn*L‐*trn*F, *trn*T‐*trn*L, *psb*D‐*trn*T		*psb*A‐*trn*H		*ycf*4‐*cem*A			*pet*B‐*pet*D	
								
Initial denaturation	5 min 95°C	
								
Initial annealing	45 s 48°C	
Initial denaturation	5 min 95°C		5 min 95°C		5 min 94°C		10 min 95°C		Initial elongation	1 min 72°C	
Denaturation	1 min 95°C	35×	1 min 95°C	35×	1 min 95°C	30x	30 s 95°C	34×	Denaturation	1 min 95°C	35×
Annealing	1 min 50°C (0.3°/s)		1 min 60°C		1 min 55°C		1 min 50°C		Annealing	45 s 48°C (2 s/cycle)	
Elongation	4 min 65°C		4 min 65°C		2 min 72°C		2 min 72°C		Elongation	1 min 72°C	
Final extension	5 min 65°C		10 min 65°C		10 min 72°C		10 min 72°C		Final extension	10 min 72°C	

### 2.4 Statistical analyses

For each species, a matrix containing complete sequences for all variable regions was prepared. We further refer to these combined datasets of selected non‐coding chloroplast regions as “haplotype(s)” and clusters of closely related haplotypes as “haplotype lineage(s).” Species with no variability in all tested regions were not included in further analyses of sequence data.

Alignments of new sequences and downloaded sequences from GenBank were made by online MAFFT v.7 (Katoh et al., 2018; Kuraku et al., 2013; https://mafft.cbrc.jp/alignment/server/) using default Auto setting and manually adjusted when necessary.

Newly obtained sequences of the taxa with published phylogeographies were evaluated only on basis of the alignment. Haplotype separation and visualization of the new datasets (i.e., *Campanula uniflora* and *Tofieldia pusilla*) were performed using R version 4.1.2 (R Core Team, 2013). Parsimony analyses to produce haplotype networks were carried out by “haplotypes” R‐package (Caner, 2020) (we used single indel coding method). NJ network was performed by “phangorn” R‐package (Schliep, 2011; Schliep et al., 2017) using hamming distance. Maps of haplotype distributions were performed using “ggplot2” (Wickham, 2016), “sp” (Pebesma & Bivand, 2005), and “rworldmap” R‐packages (South, 2011). Species distribution maps of all tested species were taken from Hultén and Fries (1986) and updated according to maps published together with their phylogeographies (Allen et al., 2012; Alsos et al., 2005; Eidesen, Alsos, et al., 2007; Gussarova et al., 2015; Marr et al., 2013; Schönswetter et al., 2007; Wang et al., 2016; Westergaard et al., 2011).

Consensus ML trees were found for all variable taxa (it means all except of *Arnica angustifolia* and *Pinguicula alpina*). For the ML phylogeny analyses, only one variable sequenced region per species was chosen and sequences of the closest dated relative taxa were downloaded from GenBank. The ML trees were time‐calibrated according to published dated phylogenies (except for *Juncus biglumis* for which any calibrated phylogeny of Cyperaceae is missing). Since we could not infer age of most recent haplotypes, the tips were set to 0. Thus, the age in the calibrated trees was underestimated. For *Arenaria humifusa*, we used *rps*16‐*trn*Q region and *Scleranthus perennis* MT094069.1 as a dated outgroup (Frajman et al., 2009). For *Bistorta vivipara* and *Oxyria digyna*, we used *psb*A‐*trn*H and *Fagopyrum dibotrys* EU554044.1 as a dated outgroup (Fan et al., 2013). For *Campanula uniflora*, we used *rpl*32‐*trn*L and *Platycodon grandifloras* NC_035624.1 as a dated outgroup (Crowl et al., 2014; Park et al., 2006). For *Silene acaulis*, we used *rpl*32‐*trn*L region and *Dianthus leucophoeniceus* OU862945.1 as a dated outgroup (Rautenberg et al., 2012, 2010). For *Tofieldia pusilla*, we used *trn*L‐*trn*F region and *Harperocallis flava* AB451606.1 as a dated outgroup (Eguchi & Tamura, 2016). For *Vaccinium uliginosum* ssp. *microphyllum*, we used *trn*L‐*trn*F region and *Andromeda polifolia* JF801624.1 as a dated outgroup (Schwery et al., 2015). For the non‐calibrated phylogeny of *Juncus biglumis*, we chose *trn*L‐*trn*F region and *Juncus castaneus* AY437954.1 as an outgroup. For all datasets, we estimated the best evolution model by ModelFinder (Kalyaanamoorthy et al., 2017) implemented in IQ‐TREE 2.1.3 (Nguyen et al., 2015). The best ML tree search was performed by IQ‐TREE 2.1.3 (Nguyen et al., 2015) with implemented fast branch test SH‐aLRT (Guindon et al., 2010), ultrafast bootstrap (Hoang et al., 2018), and with UFBoot as a protection from overestimating branch support.

## 3 RESULTS

We obtained in total 429 new sequences (GenBank: OP004939‐OP005425) originating from eight plastid non‐coding regions (Table S1).

### 3.1 Thermophilic species

**FIGURE 5:**
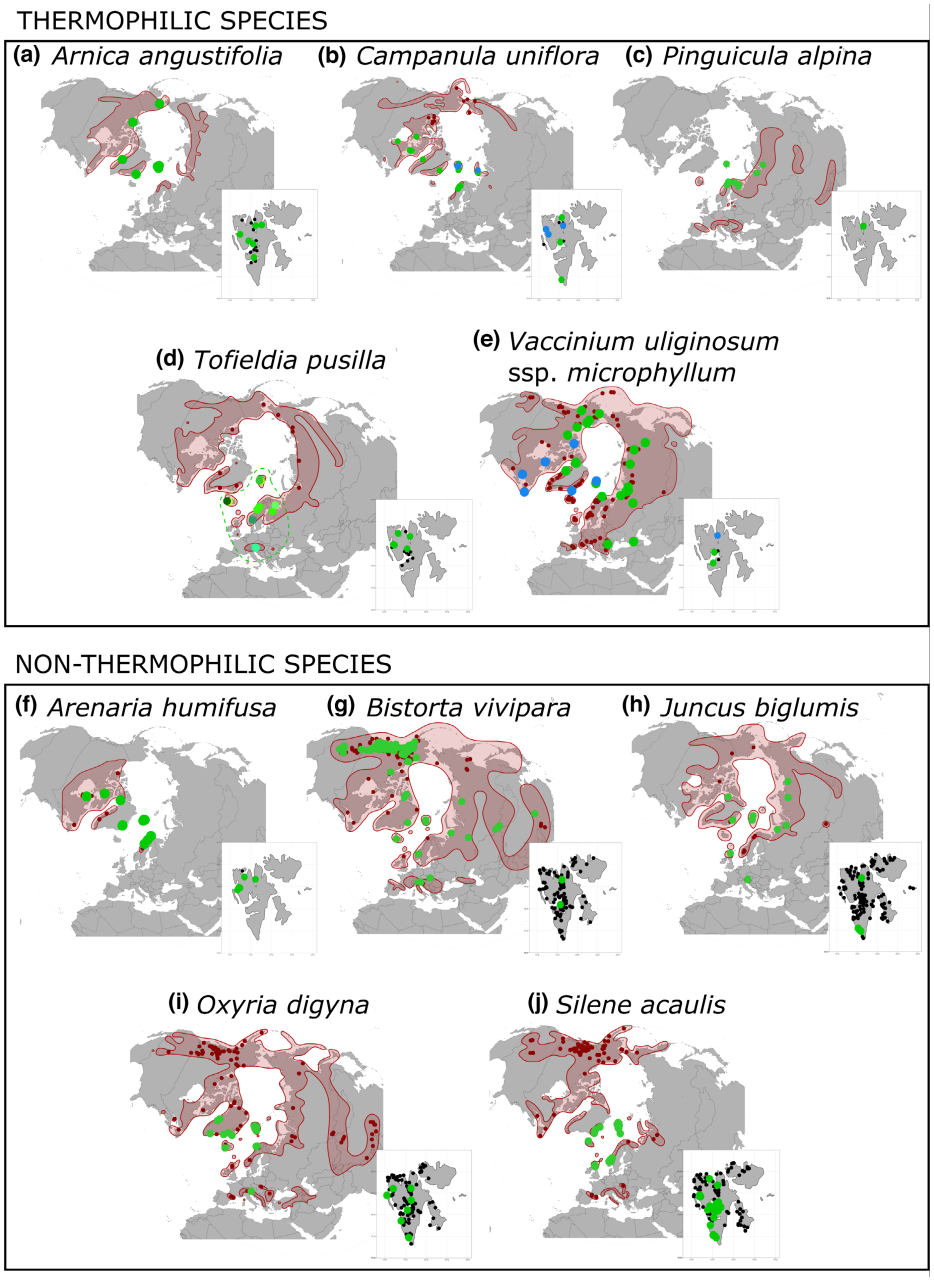
Distribution of the studied species in the Arctic and in Svalbard and total distribution of haplotype/haplotype group registered in Svalbard (NB: distribution of other haplotypes outside Svalbard omitted). (a–e) thermophilic species; (f–j) non‐thermophilic species. Red area – total distribution range in the Arctic; small black dots – total distribution in Svalbard; small dark‐red dots – sampling published in previous studies (samples of haplotypes not found in Svalbard); colored dots – colors correspond with haplotypes found in this study. Distribution of individual species was taken from Hultén and Fries (1986) and corrected according to maps published in phylogeographies (Allen et al., 2012; Alsos et al., 2005; Eidesen, Alsos, et al., 2007; Gussarova et al., 2015; Marr et al., 2013; Schönswetter et al., 2007; Wang et al., 2016; Westergaard et al., 2011).

For *Vaccinium uliginosum* ssp. *microphyllum*, already existing data from previous phylogeographies were utilized (Alsos et al., 2005; Eidesen, Alsos, et al., 2007), and only additional samples from Ringhorndalen‐Flatøyrdalen were sequenced. The formerly sequenced populations from Svalbard were all from localities in central Spitsbergen (Figure 5e) and contained widespread Arctic haplotype C (Figure S1a). The population in Ringhorndalen‐Flatøyrdalen sequenced in this study contained haplotype E, a new haplotype for Svalbard. This haplotype was documented only in eastern Canada and Greenland in the former studies (Alsos et al., 2005; Eidesen, Alsos, et al., 2007). According to dated ML tree (evolution model BIC = F81 + F), diversification of *Vaccinium uliginosum* is dated 2.87 Mya, haplotype C is dated 190 kya (with low BS support) and is ancestral to haplotype E (Figure S2d).

For *Arnica angustifolia*, *Campanula uniflora*, *Pinguicula alpina*, and *Tofieldia pusilla*, new datasets across the Arctic were gathered and analyzed. No haplotype variability was found in *Arnica angustifolia* and *Pinguicula alpina* (Figure 5a,c); the species are discussed further solely on the basis of their distribution. The new datasets for *Tofieldia pusilla* and *Campanula uniflora* were analyzed further.

**FIGURE 6:**
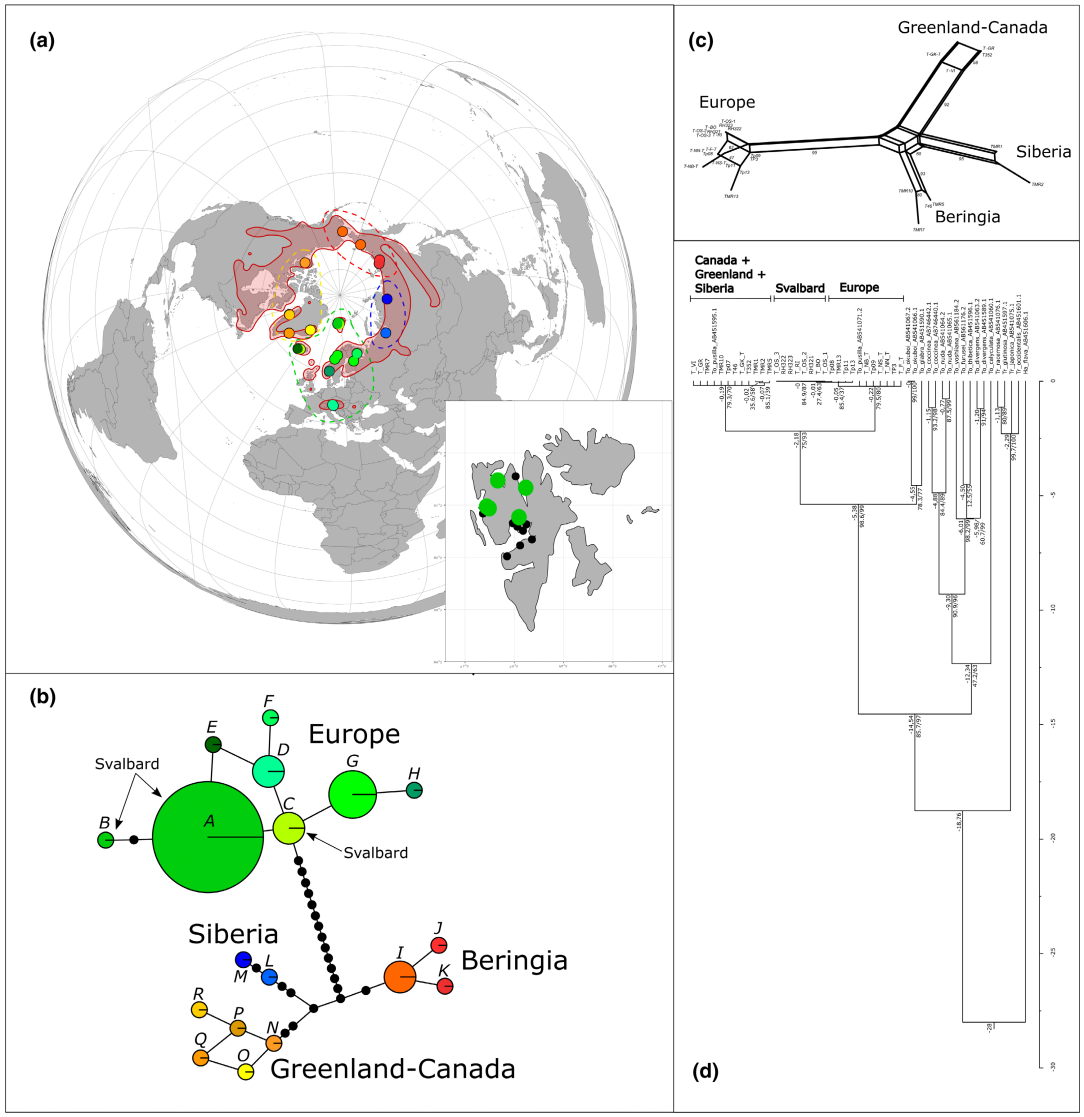
Genetic structure of *Tofieldia pusilla*. (a) Haplotype distribution in the Arctic. Red area – total distribution range in the Arctic (according to Hultén & Fries, 1986), colored dots refer to samples and specific haplotype, small black dots – total distribution in Svalbard. (b) Haplotype network of *Tofieldia pusilla* based on 4 pDNA regions and 27 variable positions including indels. Letters correspond with haplotypes are described in Table S2. Lines represent the mutational pathway connecting the haplotypes; black dots represent number of mutations between haplotypes. The size of each circle is determined by the sample size. (c) Splitnet of *Tofieldia pusilla* based on 4 pDNA regions. Calculation based on hamming distances without indel treatment. The numbers on the branches are bootstrap support values. (d) Calibrated ML tree (log‐likelihood = −2021.873) constructed from *trn*L‐*trn*F region and *Harperocallis flava* AB451606.1 as a dated outgroup (Eguchi & Tamura, 2016). Values above nodes are estimated ages of splits (in My), values below nodes are UFBoot/BS support.

In *Tofieldia pusilla*, variability was found in chloroplast regions *pet*B‐*pet*D, *trn*L‐*trn*F, *trn*S‐*trn*G, and *trn*T‐*trn*L. For these regions, 26 samples from the entire Arctic distribution were sequenced. The complete matrix of all four regions was 3572 bp long and included 27 variable positions (including indels). The nucleotide frequencies of the matrix were A = 0.3934, C = 0.1311, G = 0.1459, T = 0.3296. In total, 18 individual haplotypes were described (Table S2) in four related clusters – Europe, Beringia, Siberia, and Greenland–Canada (Figure 6a,b). All Svalbard samples belong to the European cluster. These clusters were confirmed by NJ split network (Figure 6c), ML phylogenetic tree (evolution model BIC = GTR + I + G), however, discovered only two groups (with low support) which were divided 2.18 Mya – Europe group (223.7 ky old) and Greenland‐America‐Siberia group (186.9 ky old; Figure 6d). In the tree, the Svalbard clade was well supported.

**FIGURE 7:**
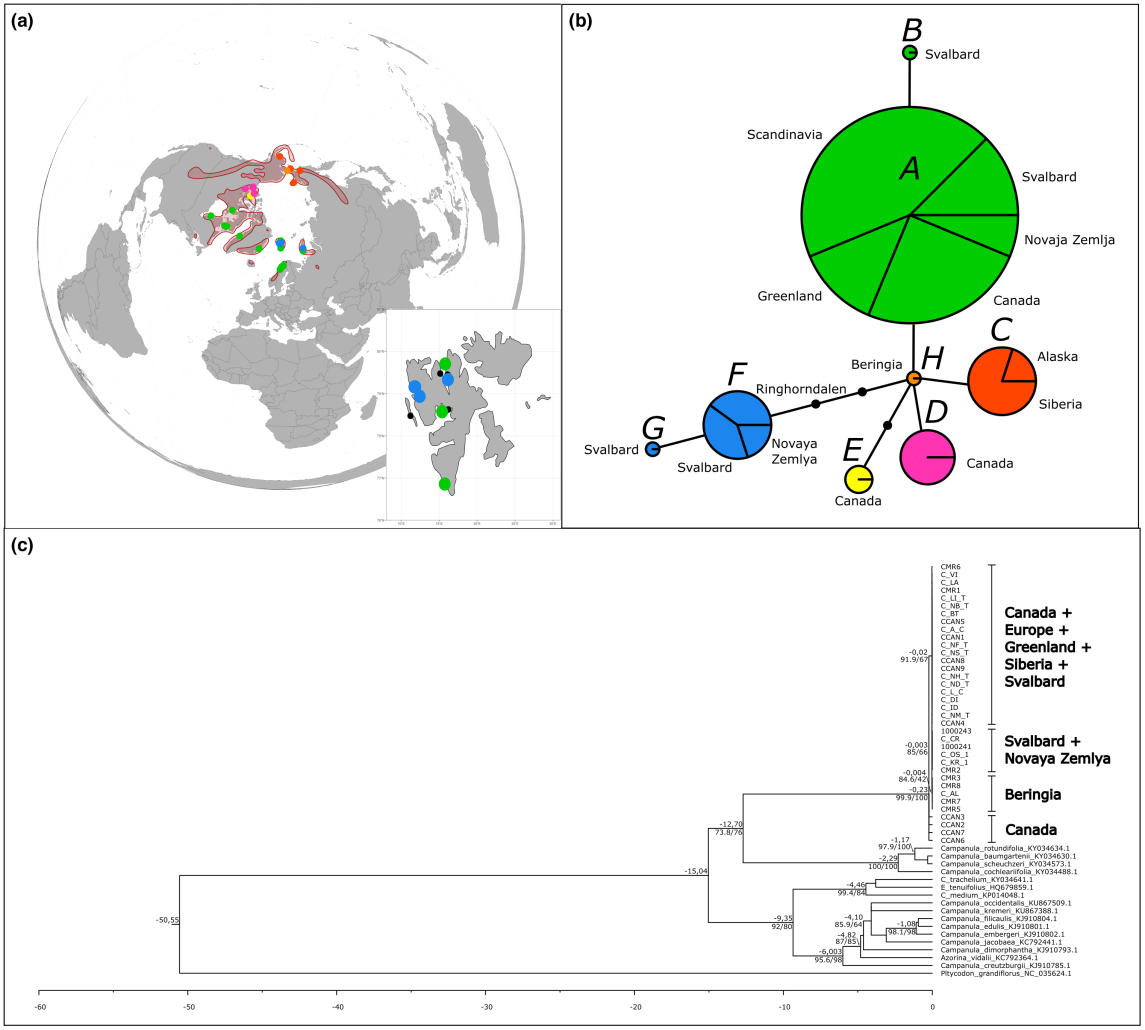
Genetic structure of *Campanula uniflora*. (a) Haplotype distribution in the Arctic. Red area – total distribution range in the Arctic (according to Hultén & Fries, 1986), colored dots refer to samples and specific haplotype, small black dots – total distribution in Svalbard. (b) Haplotype network of *Campanula uniflora* based on 4 pDNA regions and 9 variable positions including indels. Letters correspond with haplotypes described in Table S3. Lines represent the mutational pathway connecting the haplotypes; black dots represent number of mutations between haplotypes. The size of each circle is determined by the sample size. (c) Calibrated ML tree (log‐likelihood = −3924.749) constructed from *rpl*32‐*trn*L region and with *Platycodon grandifloras* NC_035624.1 as a dated outgroup (Crowl et al., 2014; Park et al., 2006). Values above nodes are estimated ages of the splits (in My), and values below nodes are UFBoot/BS support.

In *Campanula uniflora*, variability was found in regions *rpl*32‐*trn*L, *trn*L‐*trn*F, *trn*S‐*trn*G, and *trn*T‐*trn*L. For these four regions, 35 samples from the entire Arctic distribution were sequenced. The complete matrix of all four regions was 2806 bp long, and it included nine variable positions (including indels). The nucleotide frequencies of the matrix were A = 0.3457, C = 0.1609, G = 0.1575, T = 0.3359. In total, eight individual haplotypes were described (Table S3). Two haplotypes were found in Beringia, two haplotypes were found in N Canada, one haplotype was widespread in the East Canadian – Greenland – N Atlantic region (including Svalbard), and one rare haplotype lineage was restricted to Svalbard and Novaya Zemlya (Figure 7a,b). In Svalbard, this rare haplotype lineage was found in Ringhorndalen‐Flatøyrdalen and Kongsfjorden (Figure 7a). The calibrated phylogeny (evolution model BIC = TVM + F + G4) suggested a rather recent diversification of *Campanula uniflora* (Figure 7c). A 227.4 ky old haplotype from central Canadian Arctic (haplotype D) was placed at the base. Most of the Canadian/European/Greenlandic haplotypes were estimated to be 23.6 ky old. Two youngest clades (with low support) were composed of Beringian samples (haplotype C; 3.9 ky old) and samples from Svalbard and Novaya Zemlya (haplotype F + G; 3.3 ky old).

### 3.2 Non‐thermophilic species

For all non‐thermophilic species, we aligned new sequences to existing haplotype data (Allen et al., 2012; Gussarova et al., 2015; Marr et al., 2013; Schönswetter et al., 2007; Wang et al., 2016; Westergaard et al., 2011). In all cases, the new samples from Ringhorndalen‐Flatøyrdalen were aligned to the only (or the most common) haplotype present in Svalbard (Figures 5f–j, S1).

In *Bistorta vivipara*, the haplotype found in Ringhorndalen‐Flatøyrdalen was the same as registered in another location in Svalbard; this haplotype was found all around the Arctic except for NE Asia (Marr et al., 2013). The Ringhorndalen‐Flatøyrdalen population of *Juncus biglumis* was assigned to the “Arcto‐alpine” lineage, which was the most common haplotype described previously in Svalbard and in Arctic and alpine regions of the northern hemisphere (Schönswetter et al., 2007). The Svalbard populations, including Ringhorndalen‐Flatøyrdalen population, of *Oxyria digyna* contained haplotype B; this haplotype is dominating in Arcto‐alpine areas of the Northern Atlantic (Allen et al., 2012). In *Silene acaulis*, the haplotype L dominating in the Northern Atlantic area was found in Ringhorndalen‐Flatøyrdalen as well as in most of other Svalbard localities (another related European haplotype was found by previous study in Hornsund; Gussarova et al., 2015). All Svalbard samples of *Arenaria humifusa* were assigned to haplotype B, which dominates in the Arctic of Europe, Greenland, and Canada (Westergaard et al., 2011).

The calibrated phylogenies suggested that all haplotypes (except for old splits in *Tofieldia pusilla* and *Vaccinium uliginosum* ssp. *microphyllum*) were of recent origin. Svalbard haplotype of *Arenaria humifusa* was derived 12 kya (evolution model BIC = HKY + F + G4); Svalbard haplotype of *Bistorta vivipara* is very recent and haplotype of *Oxyria digyna* present in Svalbard is 90.9 ky old (both under evolution model BIC = TVM + F + I); haplotype of *Silene acaulis* existing in Svalbard is 14 ky old (evolution model BIC=K3Pu + F + G4). Topology of *Juncus biglumis* ML tree (evolution model BIC=F81 + F) is in agreement with the topology presented in Schönswetter et al. (2007).

## 4 DISCUSSION

### 4.1 No uniform colonization direction of Ringhorndalen‐Flatøyrdalen or other hotspot areas

The aim of our study was to compare directions of dispersal of thermophilic and non‐thermophilic populations in Ringhorndalen‐Flatøyrdalen and in later colonized areas in Svalbard. Results showed that thermophilic species did not colonize Ringhorndalen‐Flatøyrdalen or Svalbard in general in uniform direction of dispersal. Thus, we do not give strong evidence for a dominating direction of dispersal or dominant source region for colonization of Svalbard in early Holocene. On the contrary, our results show that thermophilic species in Ringhorndalen‐Flatøyrdalen have several source regions and different direction of dispersal, since the haplotype of *Vaccinium uliginosum* ssp. *microphyllum* found in Ringhorndalen‐Flatøyrdalen is distributed westward from Svalbard, *Tofieldia pusilla* to the south, whereas the origin of *Campanula uniflora* populations in Ringhorndalen‐Flatøyrdalen is unclear.

Further to this, our results from other locations in Svalbard also suggest that Svalbard has been colonized by thermophilic species on several occasions and from various directions. In *Vaccinium uliginosum* spp. *microphyllum*, we confirmed the presence of two haplotypes in Svalbard from two different locations. These two haplotypes have different Arctic distributions (Figure 5e). Thus, our findings support multiple colonization events from different directions in line with former findings based on AFLP data, which suggested colonization of *Vaccinium uliginosum* spp. *microphyllum* to Svalbard from both western and eastern source areas (Eidesen, Alsos, et al., 2007). Another thermophilic species *Campanula uniflora* is somewhat unclear in this respect, but presence of two independent lineages suggests also multiple colonization events; both lineages found in Svalbard probably originated from source regions west of Svalbard (discussed below; Figure 7b). Multiple colonization events were documented also in former studies. Two independent haplotype lineages arriving at Svalbard from different directions (east and south) were for example described in a rare thermophilic *Euphrasia wettsteinii* by Gussarova et al. (2012). The fact that the rare thermophilic species arrived in Svalbard from various directions implies that migration routes from all directions were available early in Holocene and had rich species pool in the HCO when the establishment conditions in Svalbard were optimal. Therefore, by that time LDD vectors from all directions might have been available to mediate transport of the propagules. Estimation of which specific vector dominated in early Holocene would be in this case a pure guess. The only dispersal vector to hotspots which was certainly not participating in Svalbard plant colonization was humans. There is no sign of human impact on the archipelago until its discovery in 1596 by Willem Barentz (Kruse, 2016), and no archeological signs of settlements were found around hotspots (Askeladden, https://www.kulturminnesok.no/).

Despite of this variability of colonization direction, most thermophilic species in Svalbard show low levels of genetic diversity, suggesting strong founder effect during colonization (Alsos et al., 2007; Birkeland et al., 2017). The low establishment and survival success has been explained by small size of Svalbard as the sink region and harsh conditions rather than by biotic factors such as competition and unavailability of a free niche (Alsos et al., 2007; Birkeland et al., 2017). Interestingly, dispersal adaptations (Table 1) are of no importance in the LDD processes (Alsos, Ehrich, et al., 2015, Nathan, 2006). Nevertheless, species traits related specifically to dependence of pollinators are shown to influence both postglacial colonization efficiency (Alsos, Ehrich, et al., 2015; Eidesen et al., 2017) and genetic loss too. A comparative study of postglacial colonization of islands in the North Atlantic showed much stronger founder effect for insect‐pollinated mixed maters than wind‐pollinated outcrossing species (Alsos, Ehrich, et al., 2015). In line with theory, the founder effect increased with dispersal distance and decreased with island size (Alsos, Ehrich, et al., 2015), which was supported in a modeling study inferring available niches for 30 species through time since LGM (Pellissier et al., 2016). Further, establishment of species typically pollinated by efficient pollinators, like bumble bees, is likely constrained by pollinator deficiency in former glaciated areas (Eidesen et al., 2017). After the HCO, the founder effect was likely enhanced by bottle‐necking due to loss of suitable localities and by genetic drift due to effective population sizes and reduced reproductive success. Thus, our revealed variation of source regions and dispersal direction among thermophilic species are probably an underestimate of the actual colonization pattern during HCO.

We assumed that the non‐thermophilic species were predisposed to establish widely throughout Holocene from various directions and source populations resulting in higher haplotype variability. On the contrary to our presumption, non‐thermophilic species appeared to have uniform (or nearly uniform) haplotype composition in Svalbard and thermophilic species are more variable. The opposite pattern of common species is probably determined by high connectivity between populations of common species and rapid and recurrent colonization by the dominating haplotype in the area of the Arctic or North Atlantic. Higher haplotype variability in rare species might be result of low establishment ability and geographical isolation. In isolated populations originated from various source regions and carrying different genetic pattern, rare haplotypes are preserved and not outcompeted by prevailing haplotypes as happened in the non‐thermophilic species (Skrede et al., 2009). Also, adaptation to local ballistic dispersal in *Campanula uniflora* and *Tofieldia pusilla* (Table 1) enhances the effect of keeping chloroplast haplotype variability conserved in smaller populations. Therefore, even though we predicted different pattern of haplotype composition of non‐thermophilic species, we argue that our suggested framework on how to test temporal shift in colonization patterns on basis of genetic information kept in rare species can be used also for other (re‐)colonized locations, when deglaciation history and historical climate records are available. Isolated populations with known timeframe of establishment keep undiluted genetic information from the colonization event and have the potential to answer a question about colonization processes better than widespread species.

The method we used (i.e., plastid haplotypes) did not provide high genetic resolution in most of the species; no level of variation was observed in *Arnica angustifolia* and *Pinguicula alpina*. This type of marker was chosen to be able to reuse available, published data, and for its ability to reconstruct maternal lineage pathways (dispersal by seeds or other propagules). Higher resolution of chloroplast signal might be provided by whole plastid sequencing or HybSeq with probes designed for large amount of selected plastid regions. Other genotyping methods or nuclear markers would provide better resolution, and they would also tell more about actual genetic diversity of population isolation, but the information about dispersal would be overshadowed by information about gene flow.

### 4.2 New phylogeographic datasets

We have presented new haplotype datasets for four Svalbard thermophilic species. Two of them, *Arnica angustifolia* and *Pinguicula alpina*, showed no haplotype variability in the sampled regions of the Arctic (Figure 5a,c). Lack of haplotype diversity in *Arnica angustifolia* may be a result of the predominantly apomictic nature of the species resulting in conserved genetic lineages (Wittzell, 1999). Facultative apomicts can harbor certain amount of genetic variability as a result of residual sexuality, but this affects mainly nuclear DNA (Hörandl & Paun, 2007; Trewick et al., 2004). Also, the observed decline of genetic variability might be ascribed to distribution to the northern latitudes. Low genetic diversity in the northern latitudes was also found in high Arctic populations of *Arabis alpina* with wide Arcto‐alpine distribution (Alsos et al., 2007; Koch et al., 2006), and in high Arctic specialist *Draba subcapitata* (Skrede et al., 2009). It was accounted to recent postglacial expansion and to easier establishment in harsh conditions and facilitation by populations with higher densities in high latitudes respectively. *Pinguicula alpina*, in contrast to *Arnica angustifolia*, is an Arcto‐alpine outcrosser pollinated by insects preferring open and wet habitats. The pollination strategy and habitat preferences could be a predisposition of high genetic differentiation between isolated areas or localities as shown in the only studied closely related alpine species *Pinguicula moranensis* (Alcalá & Domínguez, 2012). *Pinguicula moranensis*, however, is found in tropical mountains with longer vegetation history than the Arctic has. Therefore, in case of *Pinguicula alpina* the low genetic variability we observed is probably a result of postglacial expansion in the Arctic areas. Larger sampling in alpine areas of Europe and Asia might shed light on history of the species.

We revealed rather high haplotype variability in *Tofieldia pusilla* with 18 haplotypes distributed among four clearly defined geographic clusters (Figure 6a,b) – Europe, Greenland and Canada, Beringia, and Siberia. Our results are in line with previously published AFLP data suggesting strong differentiation between clusters of Europe and Greenland, although they did not include samples from Siberia and North America (Birkeland et al., 2017). The four clusters are very well separated, but a stronger genetic differentiation seems to be present between Europe and the rest of the Arctic (Figure 6c,d). The split between the European cluster and the rest of the samples was dated 2.18 Mya which suggests division before Pleistocene. These main clusters most likely persisted in different refugia during several glaciations, where European refugia were separated from other refugia possibly located in Beringia, and/or in Siberia, and/or in Arctic Canada. The data are not specific in this respect; neither networks nor calibrated phylogeny specify the ancestral population of this clade. However, based on previous knowledge, Beringia as the main Arctic refugium had the obvious potential to provide the most important shelter to this clade during Pleistocene. The further strong differentiation among non‐European lineages (Figure 6b–d) suggests recurrent fragmentation into separate smaller refugia during glacials; similar patterns of recurrent expansion were described for instance for *Saxifraga rivularis* (Westergaard et al., 2010), or *Cassiope tetragona* (Eidesen, Carlsen, et al., 2007). Survival in several separate refugia along the ice margins and fast recolonization are supported by pollen records showing presence of *Tofieldia pusilla* in early deglaciated areas (ca. 12.0–14.4 kya) in Europe as well as in America (Cushing, 1967a, 1967b; Ralska‐Jasiewiczowa & Rzetkowska, 1987; Whittington et al., 1996).

In *Campanula uniflora*, we discovered five haplotype lineages (Figure 7a,b). Calibrated phylogeny suggests that the ancestral population is found in central Canadian Arctic and has spread from there to Beringia, Siberia, and Europe. In Svalbard, we found two distinct haplotype lineages: a common haplotype lineage A + B and a rare lineage including haplotype F + G. The common lineage is widely distributed in Europe, Greenland, western Canada, and Novaya Zemlya. As pollen record suggests persistence of the species in European refugia (it has been recorded in newly deglaciated area of middle Norway 12.8–13.8 kya; Paus, 2021), we can assume that the pollen refers to the A + B common lineage which probably survived the glaciation in at least one European refugium. The rarer lineage F + G was only present in Svalbard and Novaya Zemlya (Figure 7b). This lineage has most likely originated recently (according to the calibrated phylogeny, Figure 7c) and has spread locally in the area of Svalbard and Novaya Zemlya. Due to low sampling, we cannot reject the possibility that the haplotype originated outside Svalbard and Novaya Zemlya, possibly in Canada where the haplotype diversity is the highest. Since the F + G lineage is not directly related to A + B lineage, the Canadian origin is in fact more plausible than origin in Svalbard. Spreading from Siberia is unlikely since the species distribution does not include a large part of Siberia, and there are no pollen records of the species in this area either.

### 4.3 Contribution to the previously published phylogeographies

In this study, we have utilized existing haplotype datasets for non‐thermophilic and one thermophilic taxa. Concerning non‐thermophilic species (*Arenaria humifusa*, *Bistorta vivipara*, *Juncus biglumis*, *Oxyria digyna*, and *Silene acaulis*), we found no additional haplotype variability comparing to the original studies. Also, there was no hotspot specificity found; all analyzed samples from hotspots possessed the only (or the most common) plastid haplotype in Svalbard. These haplotypes are, moreover, the most common ones in the Arctic, or in the North‐Atlantic region. The only exception from haplotype uniformity of common species was found in *Juncus biglumis* in the original publication (Schönswetter et al., 2007). The unique haplotype was found on the southwest coast (Nottinghambukta), outside the biodiversity hotspots. Persistence of a rare haplotype within a uniform population was explained by belonging to different ploidy level which is not homogenizing with other ploidy levels (Schönswetter et al., 2007). Concerning the thermophilic taxon, we extended the previously published data of *Vaccinium uliginosum* (Alsos et al., 2005; Eidesen, Alsos, et al., 2007) as discussed above.

## 5 CONCLUSION

We combined the known history of the early colonized Ringhorndalen‐Flatøyrdalen hotspot area in Svalbard with phylogeographic analyses of thermophilic and non‐thermophilic species to evaluate whether a prevailing direction of colonization was present before the cooling in mid‐Holocene, as establishment of thermophilic species is assumed to be promoted in this warmer period. Our framework suggesting rare species as a good model system to detect colonization events in specific timeframe appears to be well chosen; their haplotype diversity was conserved due to population isolation. High connectivity between local and source population of non‐thermophilic species most likely excludes rare haplotypes due to homogenization and may explain the lower haplotype variation in Svalbard among non‐thermophilic species. We found however no evidence of coherent colonization pattern or prevailing colonization direction of thermophilic species to the earliest available region in Svalbard (Ringhorndalen‐Flatøyrdalen) or to Svalbard in general. Although the thermophilic taxa *Campanula uniflora*, *Tofieldia pusilla*, and *Vaccinium uliginosum* ssp. *microphyllum* most likely colonized Svalbard during early Holocene, our results suggest that these species have arrived from different source regions, and they have dispersed and established more than once.

## AUTHOR CONTRIBUTIONS

**Viktorie Brožová:** Conceptualization (equal); data curation (lead); formal analysis (lead); funding acquisition (supporting); methodology (equal); writing – original draft (equal); writing – review and editing (equal). **Johannes S. Bolstad:** Methodology (equal). **Alexey P. Seregin:** Methodology (supporting); writing – review and editing (supporting). **Pernille B. Eidesen:** Conceptualization (equal); funding acquisition (lead); methodology (supporting); writing – original draft (equal); writing – review and editing (equal).

## CONFLICT OF INTEREST STATEMENT

None declared.

## Supporting information


Figure S1.

Figure S2.

Table S1.

Table S2.

Table S3.

## Data Availability

The dataset of DNA sequences has been publicly archived in GenBank under accessions OP004939‐OP005425.
