# Supplementary material for: From everywhere all at once: Several colonization routes available to Svalbard in the early Holocene
Source: Ecol Evol. 2023 Mar 19;13(3):e9892. doi: 10.1002/ece3.9892 (PMC10025081; doi:10.1002/ece3.9892)
Supplement: Supplementary file 1 — Figure S1. [file ECE3-13-e9892-s004.pdf]

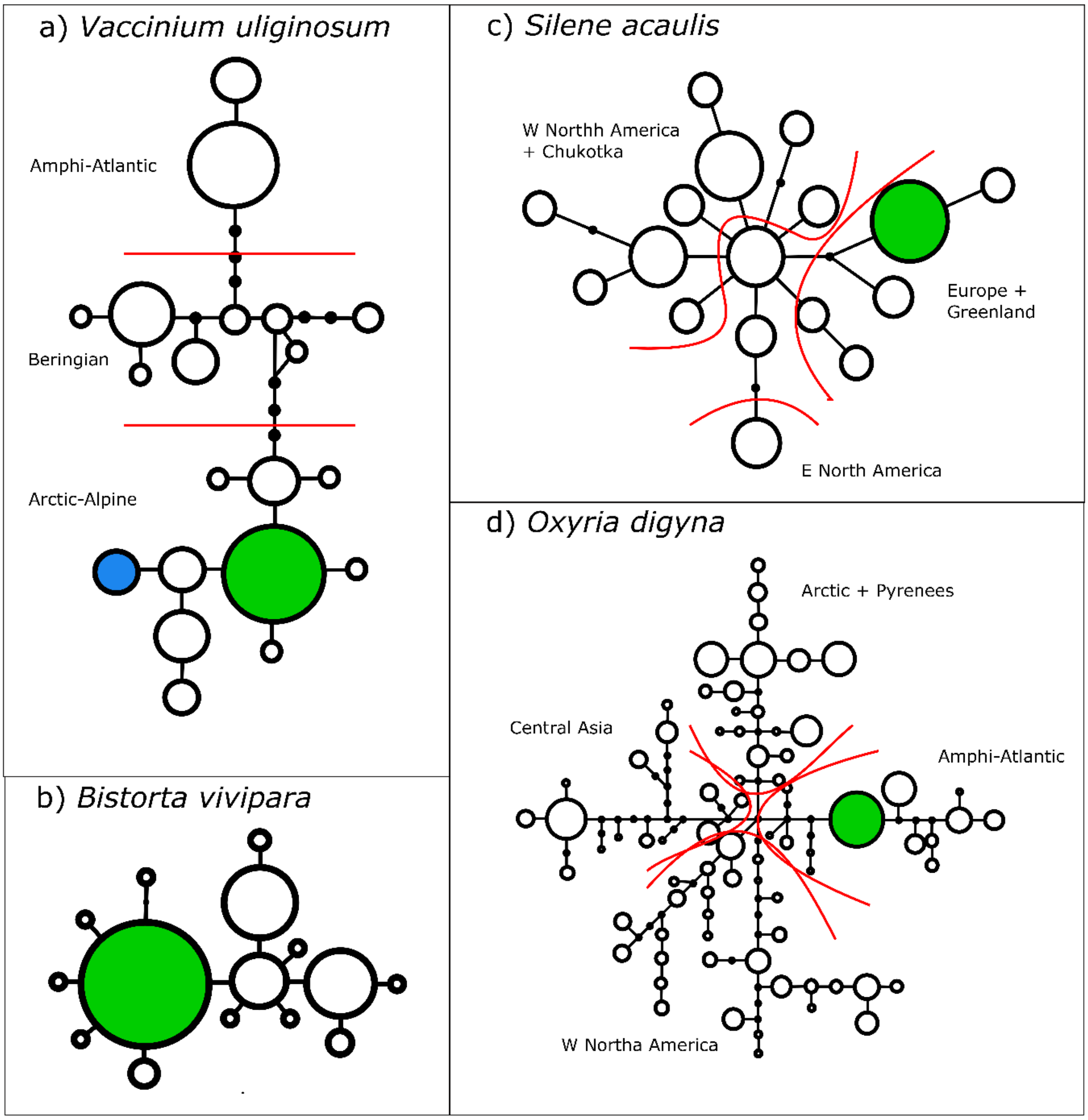

Figure S1: Haplotype networks of taxa with previously published phylogeographic datasets. All haplotype networks are adapted from the original publications (*Vaccinium uliginosum*, (Alsos et al., 2005; Eidesen et al., 2007), *Bistorta vivipara* (Marr et al., 2013), *Oxyria digyna* (Wang et al., 2016), *Silene acaulis* (Gussarova et al., 2015)). Haplotypes found in Svalbard are colored with green and blue. We do not present haplotype networks of *Juncus biglumis* and *Arenaria humifusa*, because there is low haplotype variability in the taxa, and no network was published in the original studies (Schönswetter et al., 2007; Westergaard et al., 2011).
