# Supplementary material for: From everywhere all at once: Several colonization routes available to Svalbard in the early Holocene
Source: Ecol Evol. 2023 Mar 19;13(3):e9892. doi: 10.1002/ece3.9892 (PMC10025081; doi:10.1002/ece3.9892)
Supplement: Supplementary file 2 — Figure S2. [file ECE3-13-e9892-s001.pdf]

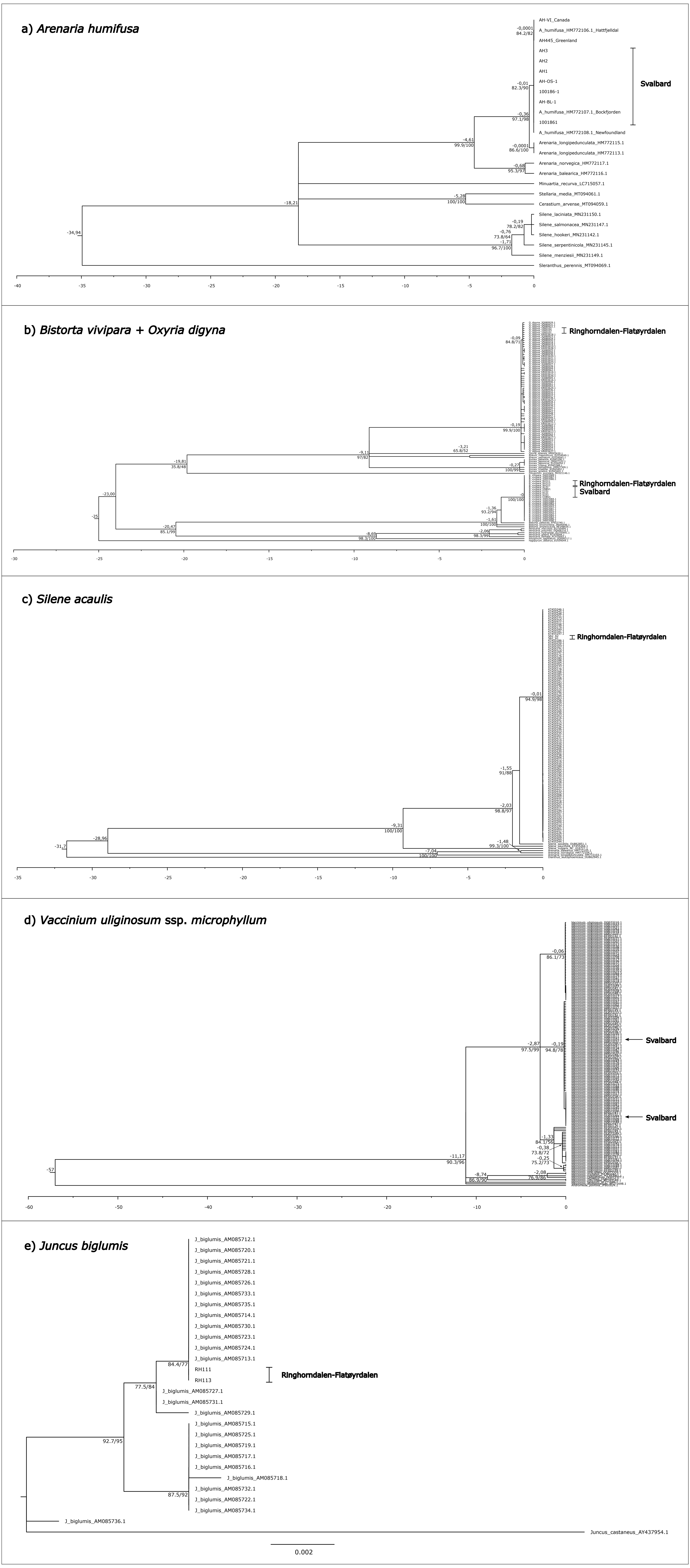

Figure S2: Calibrated consensus ML trees of *Arenaria humifusa, Bistorta vivpara, Oxyria digyna, Silene acaulis* and *Vaccinium uliginosum* ssp. *microphyllum*, and non-calibrated consensus ML tree of *Juncus biglumis*. In all calibrated trees, values above nodes is estimated age of the split (in My), values below nodes is BS support; timescale is in million years. In non-calibrated tree, age values are missing, and the bar scale shows mutation frequency. a) Calibrated ML tree of *Arenaria humifusa* ( ) constructed from *rps*16-*trn*Q region and *Scleranthus perennis* MT094069.1 as a dated outgroup (Frajman et al., 2009). Values above nodes is estimated age of the split (in My), values below nodes is BS support. b) Calibrated ML tree of *Bistorta vivipara* and *Oxyria digyna* constructed from *psb*A-*trn*H region and *Fagopyrum dibotrys* EU554044.1 as a dated outgroup (Fan et al., 2013). c) Calibrated ML tree of *Silene acaulis* constructed from *rpl*32-*trn*L region and *Dianthus leucophoeniceus* OU862945.1 as a dated outgroup (Rautenberg et al., 2012, 2010). d) Calibrated ML tree of *Vaccinium uliginosum* ssp. *microphyllum* constructed from *trn*L-*trn*F region and *Andromeda polifolia* JF801624.1 as a dated outgroup (Schwery et al., 2015). e) Non-calibrated ML tree of *Juncus biglumis* constructed from *trn*L-*trn*F region and *Juncus castaneus* AY437954.1 as an outgroup.
